# “Radix Saniculae”: Phytochemical Characterization and Potential Adulteration of an Austrian Traditional Wound-Healing Agent

**DOI:** 10.3390/plants14020266

**Published:** 2025-01-18

**Authors:** Elisabeth Eichenauer, Christina Sykora, Karin Ortmayr, Sabine Glasl

**Affiliations:** 1 Department of Pharmaceutical Sciences, Division of Pharmacognosy, University of Vienna, Josef-Holaubek-Platz 2, 1090 Vienna, Austria; elisabeth.eichenauer@univie.ac.at (E.E.); christina.sykora@univie.ac.at (C.S.); karin.ortmayr@univie.ac.at (K.O.); 2 Vienna Doctoral School of Pharmaceutical, Nutritional and Sport Sciences, University of Vienna, Josef-Holaubek-Platz 2, 1090 Vienna, Austria

**Keywords:** Austrian traditional medicine, wound-healing plants, *Sanicula europaea*, *Cardamine enneaphyllos*, phytochemical composition

## Abstract

The aerial parts (Herba Saniculae) and the underground parts (Radix Saniculae) of *Sanicula europaea* (sanicle) have been used traditionally in Austrian folk medicine to treat wounds. Interestingly, in the Austrian vernacular, “Radix Saniculae” can also refer to the underground parts of *Cardamine enneaphyllos*. This ambiguity can lead to mistakes in using these two plants and, importantly, adulterations. The present work aims to shed light on using Radix Saniculae as a wound-healing agent. Thus, the main components in the aerial and the scarcely investigated underground parts of *Sanicula europaea* were identified and compared to the underground parts of *Cardamine enneaphyllos*. For this purpose, different analytical techniques were employed: TLC, HPLC-DAD/ELSD, UHPLC-ESI-MS, and GC-MS. The main components in both *Sanicula* and *Cardamine* plant extracts were saccharides. Both parts of *Sanicula europaea* showed similar compositions: hydroxycinnamic acid derivatives and triterpene saponins. In contrast, the underground parts of *Cardamine enneaphyllos* contain two glucosinolates and their breakdown products. These findings suggest the same wound-healing activity for the underground parts of *Sanicula europaea* as was already found for its aerial parts. The glucosinolates detected in *Cardamine enneaphyllos* substantiate its use in wound healing. Nevertheless, the presented analytical methods allow for easy discovery of adulterations.

## 1. Introduction

The treatment of acute and chronic wounds benefits from multicomponent mixtures such as plant extracts, which have been used for hundreds of years in traditional medicine. The broad spectrum of plant substance classes present in extracts of a single plant leads to a multi-targeted wound healing promotion via different pathways and mechanisms. With minimal side effects, good effectiveness, ubiquitous availability, and low costs, medicinal plants represent promising and necessary treatment alternatives to prevent wounds from becoming chronic [1,2,3,4].

In Austrian folk medicine, different plants have been used for centuries to treat wounds. The valuable knowledge about traditionally used plants in Austria was compiled in the so-called VOLKSMED database [5]. Using the indication “wound healing” as a filter in this database, the most frequently referred plants are *Hypericum* sp. (*H. maculatum* and *H. perforatum*), *Arnica montana,* and *Calendula officinalis*. One of the lesser-cited plants is *Sanicula europaea* L., wood sanicle (German: Sanikel), which has not yet been investigated well concerning its wound healing potential [5,6]. Sanicle belongs to the family of Apiaceae and is native to Europe and northern Africa [7]. The aerial parts, as well as the underground parts, are mentioned in the VOLKSMED database for medicinal use. The rhizome and/or roots are traditionally prepared as an ointment using lard or butter or are extracted with oil and applied onto the wounds. The herb and/or leaves are primarily prepared as a tea to rinse and bath wounds, put directly onto the wounds, or prepared as an ointment using lard [5]. In Austria, the herb of sanicle can be purchased in pharmacies, whereas the underground parts are not commercially available. The plant is used traditionally not only for wound healing but also in the treatment of gastrointestinal hemorrhages, catarrhs, and other lung diseases [8,9]. The herb of *Sanicula europaea* is known to possess anti-inflammatory, antioxidant, antiviral, and antimicrobial activities [10,11]. Rosmarinic acid, a hydroxycinnamic acid, was found to be one of the main components in the herb of sanicle [8,10]. Besides hydroxycinnamic acids, flavonoids, saccharides, tannins, coumarins, essential oil, and triterpene saponins are described in the aerial parts [8,10,12,13]. Although in an in vitro cell assay using 3T3 fibroblasts, an extract of the herb of sanicle showed no enhancement of wound closure, it was revealed to be beneficial as an antibiotic and debridement boosting agent [14]. In contrast, the roots of sanicle have not been investigated well to date and have only been tested once in a study by Vogl et al. [15], exhibiting an in vitro anti-inflammatory effect.

**Figure 1:**
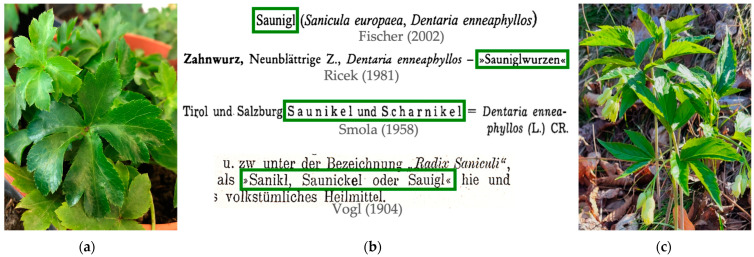
(**a**) Leaves of *Sanicula europaea* L. (Engl. wood sanicle, German: Sanikel); (**b**) examples of Austrian vernacular names of drooping bittercress from the literature (Fischer (2002) [19], Ricek (1981) [18], Smola (1958) [22], Vogl (1904) [24]); (**c**) aerial parts of *Cardamine enneaphyllos* (L.) Crantz (old synonym *Dentaria enneaphyllos* L., Engl. drooping bittercress, German: Neunblatt-Zahnwurz).

Considering the Austrian vernacular language, another plant could be meant by the German name “Sanikel”, namely drooping bittercress (Brassicaceae) [16] (*Cardamine enneaphyllos* (L.) Crantz, old synonym *Dentaria enneaphyllos* L.; German: Neunblatt-Zahnwurz) [7]. Several different vernacular names from Austria and Bavaria, Germany, for drooping bittercress can be found in the literature, all closely resembling the German name of *Sanicula europaea* “Sanikel”: “Sanigl” [17], “Saunigl” [18,19,20], “Schanikel” [21], “Scharnikel” [21,22], “Scharniggl” [23], “Sanikl, Saunickel or Sauigl” [24] (Figure 1).

Therefore, these vernacular names may lead to misinterpretation of the correct botanical source. Scientific reports from the early twentieth century describe the problem of the misleading name “Radix Saniculi/Saniculae” for the rhizome and roots of *Cardamine enneaphyllos*. In 1904, “Radix Saniculi” from drooping bittercress was equated with “Radix Saniculae” from wood sanicle, leading to intense microscopic and the first phytochemical investigations [23,24]. Even *the Austrian field flora* by Fischer et al. [17] states that “Radix Saniculae” is derived from *Cardamine enneaphyllos* instead of *Sanicula europaea*. Interestingly, the roots and rhizomes of drooping bittercress are also mentioned in the VOLKSMED database as wound-healing agents. They are prepared as an ointment with lard or butter, or the powdered roots are directly applied to wounds [5]. In 1737, the external use of drooping bittercress for wound healing was described by Nicolas Lémery [25]. Furthermore, this plant has been used to treat colic or dysentery [20]. However, the phytochemical composition of *Cardamine enneaphyllos* has not been well investigated so far. Schultz and Wagner were the first authors to describe the presence of glucosinolates in the aerial parts of the plant [26]. In 1985, Jurenitsch et al. [20] published the identification of two substances isolated from the underground parts of drooping bittercress, namely cleomin and sisymbrin, and their genuine glucosinolates glucocleomin and glucosisymbrin.

The phytochemical composition of Herba Saniculae and some bioactivities related to wound healing are already known [8,9,10,11,12,13,14]. Therefore, this work’s first aim was to compare the aerial and underground parts of *Sanicula europaea* to find differences and similarities in their phytochemical composition. Since the preparation method of the underground parts of *Sanicula europaea* in the VOLKSMED database resembles the preparation method of the roots and rhizomes of *Cardamine enneaphyllos*, the second aim of this work was to compare the composition of these two different plants, implying the development of respective chromatographic systems. The data gained are essential for quality control and for detecting potential adulterations. Furthermore, once the main constituents of all extracts were known, a literature search was conducted to identify the possible effects of these compounds contributing to a wound-healing effect. These results should shed light on the topical use of Radix Saniculae in Austrian folk medicine.

## 2. Results

### 2.1. Thin-Layer Chromatography (TLC)

**Figure 2:**
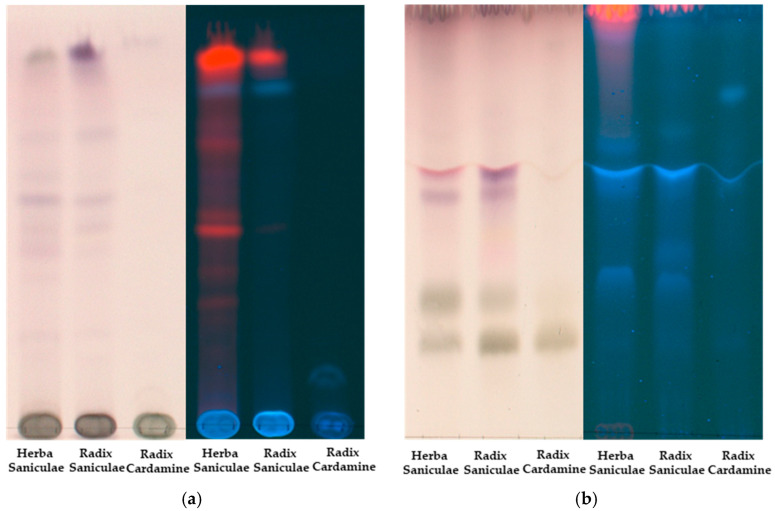
TLC comparison of the three extracts in (**a**) system A after derivatization with anisaldehyde (**left**) at visible light and without derivatization at 365 nm (**right**) and (**b**) system B after derivatization with anisaldehyde (**left**) at visible light and without derivatization at 365 nm (**right**).

A first comparison of all methanolic extracts was conducted using TLC. In the more apolar system A (Figure 2a), Radix and Herba Saniculae exhibited similar light spots, with more intensive red fluorescent chlorophyll spots in the aerial than in the underground parts. Radix Cardamines enneaphyllos required a more polar system since the sample components remained at the starting line, similar to a large fraction of unresolved compounds in both sanicle extracts. Using the more polar system B (Figure 2b), the same two spots could be found in all three extracts after derivatization with anisaldehyde/sulfuric acid: a dark green spot at Rf 0.31 and another just below, at Rf 0.23. Using pure substances, these spots were identified as disaccharides such as sucrose (Rf 0.23) and monosaccharides such as glucose and fructose (Rf 0.31). Furthermore, the extracts of *Sanicula europaea* showed similar purple spots at Rf 0.57 and Rf 0.62 after derivatization with anisaldehyde, indicating terpenoids [27]. Since it is known that sanicle contains saponins [8], another derivatization method was conducted to verify the suspicion that these purple spots were saponins. Therefore, the characteristic hemolytic activity of saponins [28] was tested using erythrocytes in human blood to make the respective spots on the TLC plate visible (Figure S1).

After applying blood gelatine onto the TLC plate and incubating for 4 to 6 h, hemolytic activity could be seen as whitish spots on the red blood gelatine. This experiment suggested that the purple spots after derivatization might originate from saponins and that this substance class might be present in both aerial and underground parts of sanicle, as already described in the literature. However, the presence of saponins had to be confirmed using further methods such as UHPLC-ESIMS. In contrast, in the extract of *Cardamine enneaphyllos*, no spots other than those of the carbohydrates were detected using this mobile phase system and anisaldehyde/sulfuric acid as the derivatization reagent. Therefore, another chromatographic technique had to be used to find other constituents.

### 2.2. High-Performance Liquid Chromatography–Diode Array Detection/Evaporative Light Scattering Detector (HPLC-DAD/ELSD) and Ultra High-Performance Liquid Chromatography–Electrospray Ionization Mass Spectrometry (UHPLC-ESIMS)

**Figure 3:**
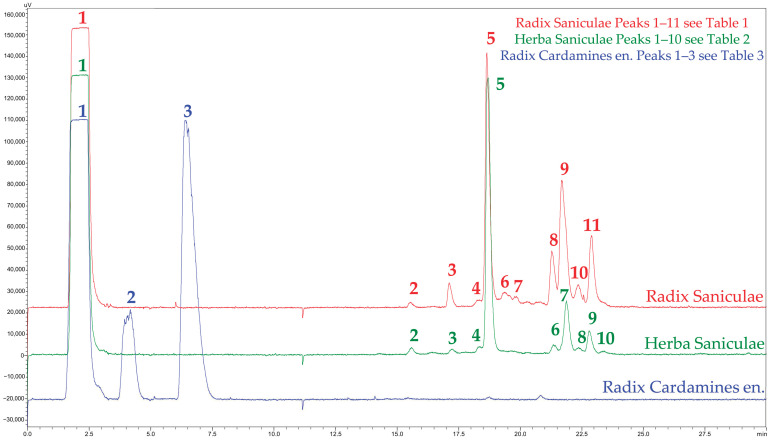
HPLC-ELSD chromatogram of all methanolic extracts (stationary phase: C18 Polar Advantage; mobile phase: water + 0.1% formic acid, acetonitrile + 0.1% formic acid). For peak identities (Peak ID) see Table 1, Table 2 and Table 3.

The next step for further characterization and identification of the main components or compound classes was an analysis via HPLC-DAD/ELSD. An HPLC method using a C18 Polar Advantage column was developed, providing broad selectivity for compounds ranging from nonpolar to moderately polar. Moreover, two different detectors were used to maximize the information gain: a diode array detector to record the UV spectra of different substances and a universal ELSD to deduce semi-quantitative information about the content of various components. From the ELSD trace, only the main components of each extract were chosen for further analysis, characterization, and putative identification (Figure 3). The TLC experiments have already suggested that all extracts contained large amounts of saccharides, which did not interact with the C18 Polar Advantage stationary phase. Consistently, the HPLC-ELSD chromatogram showed a large signal corresponding to compounds that elute in the dead volume as peak **1**, most likely different sugars. Besides these carbohydrates, the methanolic extract of *Cardamine enneaphyllos* contained only two more peaks of interest, peak **2** at Rt 4.18 min and peak **3** at Rt 6.14 min. A completely different pattern could be found for both sanicle extracts, where aerial and underground parts showed a qualitatively similar pattern. Peaks **2**, **3,** and **4** occurred as minor components, even though **3** appeared in a higher concentration in the underground than in the aerial parts. Peak **5** was the main component in Radix and Herba Saniculae extracts. In the later-eluting fraction, peaks **6**–**11** were detected in Radix Saniculae and peaks **6**–**10** in Herba Saniculae. These peaks seemed similar or related in both plant parts; however, they had a higher content in the underground than in the aerial parts.

Mass spectrometry (UHPLC-ESIMS) was chosen as a further analytical tool to gain more information about the major components detected by HPLC-ELSD. Of note, although the same method was used for the UHPLC as for the HPLC analyses, the retention times slightly deviated due to the different instrumentations used (UHPLC-CAD chromatograms see Figure S1–S3). However, the peak pattern from the UHPLC-CAD corresponded to the respective HPLC-ELSD chromatograms in all three extracts, confirming identical chromatographic performance. A mass spectrum was extracted for each prominent peak mentioned above, and mass signals were searched in SciFinder^®^ for corresponding constituents already known and published in sanicle or related family members. Annotated compounds were identified by co-chromatography with pure reference substances, if available, as suggested by the SciFinder^®^ search results. A summary of the characterization of the main components labeled by numbers in the HPLC-ELSD chromatogram (Figure 3) of each extract can be found in Table 1 and Table 2 for the extracts of *Sanicula europaea* and Table 3 for the extract of *Cardamine enneaphyllos* (for the respective UV and mass spectra, see Tables S1–S3). In cases where pure substances for final identification were unavailable, the detected constituents are given as belonging to a “compound class” or are indicated as “putatively assigned” (Table 1, Table 2 and Table 3), implying that further fractionation and purification of components for structure elucidation must be realized in future work.

**Figure 4:**
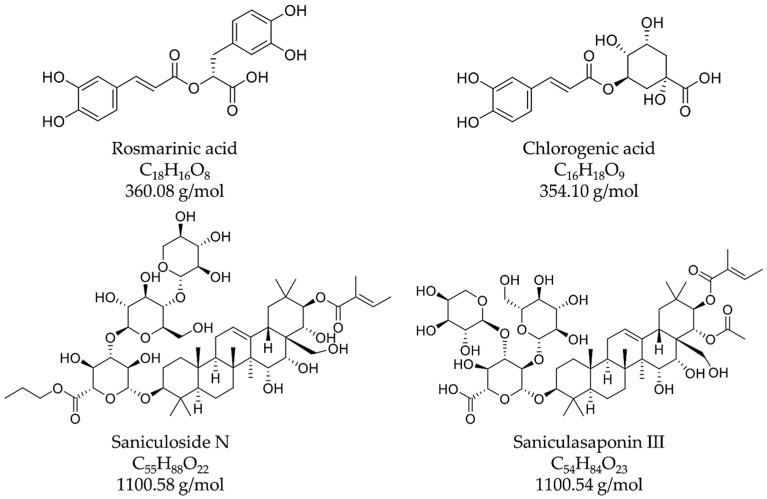
Structural and molecular formulas and monoisotopic masses of the hydroxycinnamic acid derivatives rosmarinic acid (**upper left**) and chlorogenic acid (**upper right**) and the saponins saniuloside N (**lower left**) and saniculasaponin III (**lower right**).

As the prior analyses suggested, the first eluting peak **1** consisted of saccharides such as sucrose (CAS: 57-50-1). They showed no interaction with the stationary phase and rushed through the system after injection in the dead volume. In both sanicle extracts, these sugars represented the main components. Besides these carbohydrates, the extracts of the aerial parts and the underground parts contained two main substance classes: hydroxycinnamic acid derivatives (peaks **2**–**5**) and saponins (peaks **6**–**11**). The main component in both extracts was peak **5**, which could be identified as rosmarinic acid (CAS: 20283-92-5, Figure 4). Furthermore, peak **2** consisted of chlorogenic acid (CAS: 327-97-9) in Herba and Radix Saniculae (Figure 4). A literature search using SciFinder^®^ suggested 4-O-ß-D-glucopyranosyl rosmarinic acid with a molecular weight of 522 g/mol as a possible substance detected as peak **3** in Radix Saniculae. Consistent with this suggestion, signals were detected at *m*/*z* values of 521.40 [M-H]^−^ and 523.16 [M+H]^+^, indicating a molecule with the matching molecular weight. Furthermore, signals matching the *m*/*z* values of rosmarinic acid and glucopyranose were detected at the same retention time, likely originating from hydrolysis during the ionization process in the MS analysis. In Herba Saniculae, peak **3** seemed to correspond to a rosmarinic acid derivative, since, again, the *m*/*z* values of rosmarinic acid and glucopyranose were detected. However, this derivative appeared to have a molecular weight of 540 g/mol according to the *m*/*z* values observed in the positive and negative mode, respectively, suggesting a hydroxylated rosmarinic acid derivative. Again, further fractionation, purification, and structure elucidation of the substance corresponding to peak **3** must be conducted to identify the respective rosmarinic acid derivative. Peak **4** in both extracts was identified as 3,4-dicaffeoylquinic acid (CAS: 14534-61-3).

As already observed in TLC analysis, the other substance class contained in both parts of sanicle was triterpene saponins. In both extracts, substances with a molecular weight of 1100 g/mol were detected, likely corresponding to one or more already-published saponins like saniculoside N or saniculasaponin III [10,29]. The other detected masses in sanicle were also likely saponins due to their molecular weight (910 g/mol, 926 g/mol, 968 g/mol, 970 g/mol, and 1102 g/mol) and their presence in the purple, hemolytic spot. However, they could not be assigned, necessitating further fractionation, purification, and structure elucidation for final identification.

**Figure 5:**
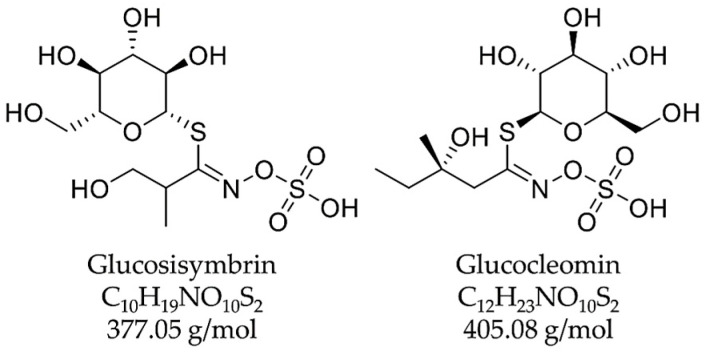
Structural and molecular formulas and monoisotopic masses of the glucosinolates glucosisymbrin (**left**) and glucocleomin (**right**).

Besides the already-known carbohydrates in peak **1**, two main components were putatively found in the underground parts of *Cardamine enneaphyllos*: the glucosinolates glucosisymbrin as peak **2** and glucocleomin as peak **3** (Figure 5). The detected molecular ion peaks (*m*/*z* 376.26 [M-H]^−^ and 378.24 [M+H]^+^ for glucosisymbrin; *m*/*z* 404.31 [M-H]^−^ and 406.25 [M+H]^+^ for glucocleomin) fitted with the information on these glucosinolates published by Jurenitsch et al. in 1985 [20]. To verify the presence of glucosisymbrin and glucocleomin in the extract, GC-MS was used to search for their breakdown products, sisymbrin and cleomin.

### 2.3. Gas Chromatography–Mass Spectrometry (GC-MS)

UHPLC-ESIMS analysis showed the possible presence of the glucosinolates glucosisymbrin and glucocleomin. Therefore, a GC-MS analysis was conducted with a dichloromethane (DCM) extract of the fresh, cut underground parts. In intact plant tissue, glucosinolates are separated from enzymes called myrosinase. Whenever the plant cells are destroyed, for example, by chewing or cutting, the enzyme hydrolyzes the glucosinolates, which leads to different breakdown products, such as isothiocyanates, thiocyanates, or nitriles [30]. These products can be easily analyzed using gas chromatography. Therefore, identifying the breakdown products (sisymbrin and cleomin) was crucial for determining the genuine glucosinolates.

**Figure 6:**
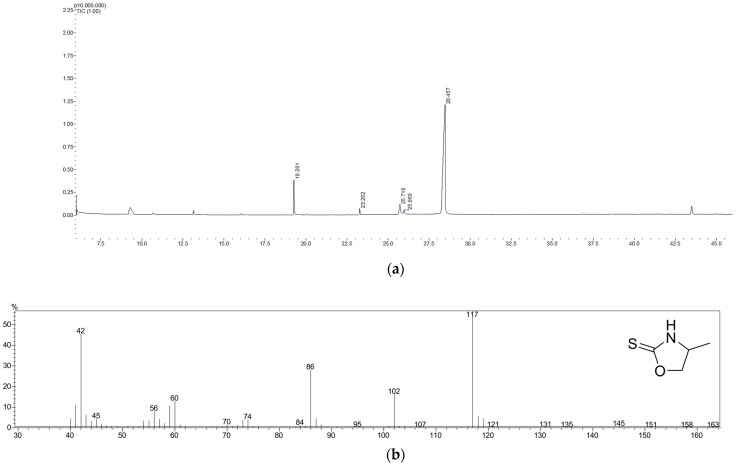

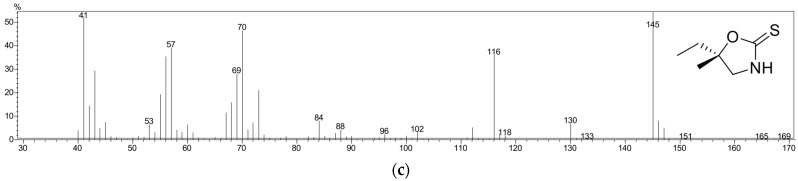
(**a**) Total ion chromatogram of the GC-MS analysis of the DCM extract of the fresh, cut underground parts of *Cardamine enneaphyllos* (sisymbrin at 25.72 min; cleomin at 28.46 min). Mass spectra and structural formula of (**b**) sisymbrin and (**c**) cleomin.

The total ion chromatogram is depicted in Figure 6a; sisymbrin eluted at 25.72 min, and cleomin eluted at 28.46 min. The mass spectra found for sisymbrin and cleomin matched those recorded by Jurenitsch et al. [20]: sisymbrin shows a base peak at *m*/*z* 117 and a fragment at *m*/*z* 102, which corresponds to the cleavage of a methyl group (Figure 6b and Table S4). Cleomin has a base peak at *m*/*z* 145, and eliminating a methyl and an ethyl group leads to fragments at *m*/*z* 130 and *m*/*z* 116 (Figure 6c and Table S4).

## 3. Discussion

The aim of this work was the phytochemical characterization of methanolic extracts of Radix and Herba Saniculae, as well as Radix Cardamines enneaphyllos. All three extracts contained compounds with a potential bioactivity related to wound healing, albeit that they partially belong to different compound classes and act through different mechanisms. Commonalities in the chemical composition suggest similar bioactivities related to wound healing. The herb of *Sanicula europaea* has been tested in an in vitro wound-healing assay on fibroblasts. In this assay, sanicle showed cytotoxic effects on cells along the wound margin and, therefore, might promote wound healing due to wound debridement and acting as an antibiotic agent. However, the responsible substances have not been investigated in this study [14].

The only chemical similarity between all three extracts was the high amount of carbohydrates, such as sucrose, glucose, and fructose. Sugars are well known for their wound-healing potential, e.g., as medicinal honey. An osmotic gradient is created when high sugar concentrations are applied to a wound. This leads to a fluid flow from subdermal tissue, which cleans the wound from debris and microorganisms. Furthermore, sugars have a low water activity, inhibiting the growth of bacteria in the wound [31]. High carbohydrate contents might, therefore, equip the extracts with antibacterial and debriding properties, which are beneficial in the early stages of wound healing.

Besides these carbohydrates, the aerial and underground parts of sanicle are composed very similarly. Two main substance classes were identified: hydroxycinnamic acid derivatives, with rosmarinic acid as the main component in both plant parts, and triterpene saponins. These findings correspond with the already published data for the aerial parts: as mentioned above, hydroxycinnamic acids, flavonoids, saccharides, tannins, coumarins, essential oil, and triterpene saponins have already been found [8,10,12,13]. However, the underground parts have not yet been phytochemically investigated. Rosmarinic acid and chlorogenic acid were already described as constituents of *Sanicula europaea* [8,11], whereas 4-O-β-D-glucopyranosyl rosmarinic acid has only been found in *S*. *lamelligera* [32]. 3,4-Dicaffeoylquinic acid or derivatives with other substitution patterns have not yet been identified in the genus *Sanicula*, but they have been identified in other genera in the family of Apiaceae, such as *Peucedanum ostruthium*, which is also used as wound-healing agent in traditional medicine [33]. The detected hydroxycinnamic acid derivatives possess antibacterial, antioxidative, and anti-inflammatory activities, which benefit wound healing [3,34,35,36,37,38]. Küba et al. [39] compared the in vivo wound-healing effect of a 10% rosmarinic acid cream to a 5% dexpanthenol group on wounds in Wistar albino rats. This study resulted in a significantly higher wound size reduction in the rosmarinic acid group than in the dexpanthenol and control group, even though there was less epithelization detected. Further studies are necessary to fully understand the molecular targets of rosmarinic acid in the complex wound healing mechanism. An ointment containing chlorogenic acid accelerated wound healing by proliferative, antibacterial, and antioxidant effects in infected wounds in diabetic rats [38]. Moreover, Chen et al. [40] showed an in vivo wound healing boosting effect of a 1% chlorogenic acid ointment due to increased collagen synthesis and antioxidant activity on excision wounds in Wistar rats. These studies presented the beneficial effects of chlorogenic acid in all stages of the wound healing mechanism, e.g., the antimicrobial effects against bacteria such as methicillin-resistant *Staphylococcus aureus*, fibroblast proliferation, and influencing the levels of different cytokines and growth factors, such as TNF-α or TGF-β [38,40].

The second substance class found in the aerial parts and in the roots and rhizomes of *Sanicula europaea* was triterpene saponins. Molecular ion peaks at *m*/*z* 1099 [M-H]^−^ and 1101 [M+H]^+^ were detected in both parts of sanicle, indicating a saponin with a molecular weight of 1100 g/mol. Saniculoside N, a triterpene saponin glycoside with anti-HIV activity, has already been isolated from the aerial parts of *Sanicula europaea* [10] and, therefore, might be one of the detected molecular ion peaks found in this work. Moreover, several other saponins with a molecular weight of 1100 g/mol have been identified in another *Sanicula* species, namely in *S*. *elata* var. *chinensis*: saniculasaponin II, III, and IV [29]. The other detected saponins could not be assigned to any published substances in the genus *Sanicula*. Nevertheless, the closely related genus *Eryngium* in the family Apiaceae contains triterpene saponins, including saniculasaponin II and III [41]. Various saponins were already published for different *Eryngium* species, some of which might match the detected [M-H]^−^ peaks at *m*/*z* 925, 928, and 910 in this work [42]. These saponins are structurally closely related to those already published for *Sanicula*, with barrigenol A_1_ or R_1_ as aglycon [41,42,43,44]. Therefore, the saponins in *Eryngium* and *Sanicula* are derivatives of oleanane-type pentacyclic triterpenes. Barrigenol-like triterpenoids are known to exhibit anti-inflammatory and antimicrobial activity [45]. Oleanane-type triterpene saponins like glycyrrhizin have already been investigated in different in vitro wound healing assays, which underline the potential of this substance class as a wound-healing agent [46]. However, the presented phytochemical characterization of extracts facilitates further isolation and structure elucidation to identify hydroxycinnamic acids and saponins as the next step.

Furthermore, the use of the roots and rhizomes of *Cardamine enneaphyllos* instead of *Sanicula europaea* in Austrian folk medicine was investigated. On the one hand, the Austrian vernacular name “Saunigl” might be derived from the German word for hedgehog, “Igel”, due to the quill-like appendages found on the rhizome of drooping bittercress [18]. Therefore, the morphological appearance and colloquial language might have been the reason for the misleading use of the term “Radix Saniculae”. On the other hand, the chemical composition of the underground parts of *Cardamine enneaphyllos* has not yet been intensively investigated. Jurenitsch et al. [20] isolated glucosinolates and their breakdown products while hunting for alkaloids. However, members of the Brassicaceae family also contain hydroxycinnamic acids and flavonoids, which were already described for *Sanicula europaea* [8,47]. Therefore, not only the vernacular term but also commonalities in the phytochemical composition could explain the use of different botanical sources for “Radix Saniculae”.

Yet, comparing the phytochemical composition, it became clear that only the high sugar content was similar between these two plants. However, even though carbohydrates are beneficial in wound healing, as mentioned above, the VOLKSMED database cites fat- or oil-based preparations for the use of “Radix Saniculae”. Therefore, the role of sugars in these wound-healing applications might be negligible. But even if carbohydrates play a minor part in the wound-healing effect of Radix Cardamines enneaphyllos, the other detected substances might also reveal promising wound-healing activities. Glucosinolate breakdown products are well known for their anticancer effect but also possess potent antioxidant and antimicrobial activities, which play an essential role in wound healing [48]. The actual wound-healing potential of these substances still has to be discovered. The hydrolytic product cleomin has been investigated regarding its antinociceptive bioactivity mediated by muscarinic and GABA_B_ receptors [49]. The literature on the bioactivities of sisymbrin is missing. However, examining the effects of these compounds is crucial for substantiating the use of drooping bittercress in wound healing and will be anticipated in future work. Even though the resulting bioactivities of the application of sanicle roots or drooping bittercress roots might differ due to the differences in their phytochemical composition, both plants contain substance classes that can benefit wound healing via antioxidant, anti-inflammatory, or antimicrobial properties.

In order to shed light on the actual wound-healing potential of both plants, the next steps will be the final isolation and identification of the main components of each extract. Furthermore, these extracts and pure substances will be tested in different in vitro assays covering bioactivities essential in wound healing (re-epithelialization of keratinocytes, antioxidant potential by inducing NRF2-signaling, or anti-inflammatory effects via inhibition of Nf-κB pathways after TNF-α stimulation). Yet, the established methods for phytochemical profiling of both plants represent basic requirements for further analysis of constituents and bioactivities.

## 4. Materials and Methods

### 4.1. Plant Material and Extraction

Herba Saniculae was supplied by KOTTAS PHARMA GmbH (200 g, W20201762, Wien, Austria). The dried roots of sanicle were also provided by KOTTAS PHARMA GmbH (wild harvesting in Slovakia, July 2022). The rhizomes and roots of *Cardamine enneaphyllos* were collected in April 2024 at Guglzipf, Berndorf, Lower Austria, Austria (47,93998° N, 16,11343° E), and identified by Elisabeth Eichenauer. Altogether, 134.1 g of underground parts were collected. The roots and rhizomes were ridden from dirt using a brush. A total of 15.2 g were directly stored at −20 °C for GC-MS analysis. The rest of the underground parts were washed with water to eliminate the remaining dirt, broken into smaller (1–3 cm long) pieces, and put into a rack dryer at 35 °C for 48 h. The dried rhizomes yielded 29.1 g, which corresponds to a drying loss of 75.6%.

The underground parts of *Sanicula europaea* and *Cardamine enneaphyllos* were comminuted using an impact mill (IKA M20 Universal mill, IKA-Werke GmbH, Staufen, Germany) and proceeded without putting them through a sieve. The consistency of the powder resulting from *Cardamine enneaphyllos* was fine-grained and looked like ashes, in contrast to *Sanicula europaea*, where the powder was more fibrous. The aerial parts of *Sanicula europaea* were already purchased as a cut herbal drug. A total of 10.0 g of each plant part was suspended in 100 mL methanol (MeOH) and extracted for approximately 2 h at room temperature on a shaker. The resulting extracts were filtered and evaporated using a Heidolph Hei-VAP rotary evaporator (Heidolph Scientific Products GmbH, Schwabach, Germany) at 40 °C. The extracts were re-dissolved in MeOH to final concentrations of 10 mg/mL (TLC) or 5 mg/mL (HPLC/MS) and centrifuged for analysis via different chromatographic techniques. For comparison of the extracts via different chromatographic techniques, the extracts of *Sanicula europaea* were called “Radix Saniculae” for the underground parts and “Herba Saniculae” for the aerial parts, and the extract of the roots and rhizomes of *Cardamine enneaphyllos* got the name “Radix Cardamines enneaphyllos”.

For GC-MS analysis, the fresh rhizomes of *Cardamine enneaphyllos* were used. After collection, they were stored at −20 °C. To activate the enzyme myrosinase by the destruction of cellular integrity and gain the volatile degradation products of glucosinolates, 5 g of the rhizome were cut into small pieces with a knife and extracted with 50 mL dichloromethane (DCM) for approximately 1 h on a shaker at room temperature. After drying with anhydrous sodium sulfate and filtering the extract, the solvent was evaporated using a Heidolph Hei-VAP rotary evaporator (Heidolph Scientific Products GmbH, Schwabach, Germany) at 40 °C. The resulting extract was re-dissolved in 100 µL DCM, centrifuged, and diluted 1:50 with DCM before GC-MS analysis. All solvents used for extraction and chromatographic analysis were bought as analytical-grade solvents from VWR GmbH (Vienna, Austria).

### 4.2. Thin-Layer Chromatography (TLC)

For TLC experiments, Silica 60 F254 TLC plates (average particle size 9.5 to 11.5 μm, aluminum sheets with fluorescence indicator, Merck KGaA, Darmstadt, Germany) were used as a stationary phase. For analysis, 10 μL of each extract (10 mg/mL in MeOH) was applied. Two different mobile phase systems were used: system A consisting of DCM-MeOH (9+1) and system B consisting of DCM-MeOH-10% formic acid (8+5+1). The CAMAG TLC Visualizer (CAMAG, Muttenz, Switzerland) was used to photograph and interpret the developed and dried plates under white light and at wavelengths of 254 nm and 366 nm. For improved visibility of the constituents, the plates were derivatized with anisaldehyde/sulfuric acid, which was applied using the CAMAG Chromatogram Immersion Device (III) (CAMAG, Muttenz, Switzerland).

To detect hemolytic triterpene saponins, TLC plates were derivatized with blood gelatin. For this purpose, 5 gelatin sheets (approx. 9 g) of “Dr. Oetker Gelatine” (A07756/03) were left to swell in water for 30 min at room temperature. Afterward, the sheets were removed from the water, squeezed, and blended in 115 mL phosphate buffer pH 7.4 as described in the Pharmacopoeia Europaea (250 mL KH_2_PO_4_-solution (0.2 mol/L) + 393.4 mL NaOH-solution (0.1 mol/L); Ph.Eur. number 11.0/4004800). This mixture was heated to 40 °C until the gelatin was completely dissolved. After that, 4 mL of human blood was added to 50 mL of buffer gelatin. The human blood was freshly drawn from an adult volunteer by a physician and stored at 4 °C before being used for the TLC experiments. Finally, the blood gelatin suspension was filtered through cotton before approximately 5 mL was applied using a graduated pipette onto the developed TLC plate, which was framed with adhesive tape to form a tray. The suspension was distributed equally on the plate and left for 4–6 h of development time at room temperature. The final plates were photographed and compared to plates derivatized with anisaldehyde/sulfuric acid to identify the hemolytic spots and their corresponding behavior after reaction with anisaldehyde.

### 4.3. High-Performance Liquid Chromatography—Diode Array Detection/Evaporative Light Scattering Detector (HPLC-DAD/ELSD)

A Shimadzu instrumentation (Degasser DGU-20A 5, Auto Sampler SIL-20AC HAT, Liquid Chromatograph LC-20AD, Column Oven CTO-20AC, Diode Array Detector SPD-M20A, Low Temperature Evaporative Light Scattering Detector ELSD-LT, Communications Bus Module CBM-20A, Lab Solutions Software, Shimadzu, Kyoto, Japan) was used for HPLC analysis. An RP18 polar advantage column (Acclaim^TM^ Polar Advantage II, C18, 3 μm, 150 mm × 2.1 mm) was used as the stationary phase, and the mobile phase was composed of water + 0.1% formic acid (A) and acetonitrile + 0.1% formic acid (B). Gradient elution was utilized at a flow rate of 0.3 mL/min as follows: 0–5 min plateau at 5% B, 5–8 min from 5 to 20% B, 8–13 min from 20 to 45% B, 13–25 min 45–70% B, followed by 5 min at 98% B and 10 min of re-equilibration at 5% B. As detectors, a DAD as well as an ELSD were used. For analysis, 5 μL of each extract dissolved to a final concentration of 5 mg/mL in MeOH were injected.

### 4.4. Gas Chromatography–Mass Spectrometry (GC-MS)

For the analysis of volatile compounds in the underground parts of *Cardamine enneaphyllos*, a Shimadzu instrumentation (GC-2010, coupled with a GCMS-QP2010 single quadrupole and an AOC-20s Autosampler; GCMS Solutions Software 4.11) was employed. As a stationary phase, an OPTIMA-5 capillary column (Macherey-Nagel^TM^, 50 m × 0.25 mm × 0.5 µm, Item number: 726099.50) was used, and the carrier gas was helium (ALPHAGAZ^TM^ 1 He purity > 99.99%; flow rate 1.14 mL/min). After splitless injection of 2 µL of samples at 270 °C, a temperature gradient was applied as follows: from 50 °C to 270 °C with 6 °C/min, followed by an isothermic plateau at 270 °C for 10 min. The scan area for the EI-QP (ion source temperature 250 °C) was set to 40–500 *m*/*z*. For the identification of substances, the spectra were analyzed using the GCMS Solutions Software 4.11 and compared with library databases (Wiley 229, NIST 27, 147) and already-published GC-MS spectra.

### 4.5. Ultra High-Performance Liquid Chromatography–Electrospray Ionization Mass Spectrometry (UHPLC-ESIMS)

An LTQ XL ion trap mass spectrometer was coupled to a Dionex UltiMate 3000 system (Thermo Fisher Scientific, Waltham, USA) and used to further characterize the main components. For separation via UHPLC, the same method and parameters as for HPLC-DAD/ELSD experiments were employed. A HESI source (300 °C heater temperature, 40/10/1 arb. units for the sheath, aux, and sweep gases, respectively, and 3.5 kV spray voltage at 275 °C capillary temperature) was used to achieve negative and positive ion mode ionization. The scans were performed with an *m*/*z* range from 100 to 1500 in positive mode and 110 to 2000 in negative mode. For data acquisition and evaluation, the Xcalibur software was utilized.

## 5. Conclusions

To conclude, the entries found for “Radix Saniculae” in the VOLKDMED database cannot be attributed to the correct botanical source with certainty. The underground parts of *Sanicula europaea* contain the same major substance classes as the aerial parts, which substantiates the traditional use of this plant part due to the already-known wound-healing effects of Herba Saniculae. However, using *Cardamine enneaphyllos* instead of sanicle due to vernacular names still holds beneficial properties in wound healing. Since “Radix Saniculae” is still used as a wound-healing agent in Austria, especially in rural areas, the established TLC and HPLC methods deliver a fast and easy way to determine the botanical source. Future research will concentrate on the isolation and structural elucidation of the principal components and bioactivity testing to further explain the traditional use of these two plants in wound healing.

## Figures and Tables

**Table 1:** Tentative annotation of main components and compound classes found in Radix Saniculae using HPLC-DAD/ELSD and UHPLC-ESIMS.

Peak ID ^1^	Rt. [min] ^1^	UV max [nm] ^1^	*m*/*z* [neg.]	*m*/*z* [pos.]	Putatively Assigned Compound/Compound Class
1	2.29	nd	341.31 [M-H]^−^, 387.27 [M+FA-H]^−^, 683.42 [2M-H]^−^, 729.20 [2M+FA-H]^−^, 1025.27 [3M-H]^−^, 1071.15 [3M+FA-H]^−^	365.37 [M+Na]^+^, 381.30 [M+K]^+^, 707.37 [2M+Na]^+^, 723.15 [2M+K]^+^	Sucrose ^2^ (sugars)
2	15.55	203, 243, 326	191.15 [M-H]^−^ Quinic acid, 353.27 [M-H]^−^, 707.34 [2M-H]^−^, 1061.12 [3M-H]^−^	355.24 [M+H]^+^, 377.32 [M+Na]^+^	Chlorogenic acid ^2^
3	17.12	200, 329	161.09 [M-H]^−^ Glucopyranose, 359.36 [M-H]^−^ Rosmarinic acid, 521.40 [M-H]^−^, 1043.39 [2M-H]^−^	163.05 [M+H]^+^ Glucopyranose, 361.11 [M+H]^+^ Rosmarinic acid, 523.16 [M+H]^+^, 721.12 [2M+H]^+^ Rosmarinic acid, 1045.06 [2M+H]^+^	4-O-ß-D-glucopyranosyl rosmarinic acid (or another rosmarinic acid derivative)
4	18.36	203, 241, 326	353.33 [M-Caffeoyl-H]^−^, 515.35 [M-H]^−^	499.38 [M-H_2_O+H]^+^, 517.20 [M+H]^+^	3,4-Dicaffeoylquinic acid ^2^
5	18.65	196, 212, 329	359.29 [M-H]^−^, 719.32 [2M-H]^−^, 1079.05 [3M-H]^−^	361.14 [M+H]^+^, 721.13 [2M+H]^+^, 1081.07 [3M+H]^+^	Rosmarinic acid ^2^
6	19.37	202, 328	1099.87 [M-H]^−^	1101.49 [M+H]^+^	Saponins
7	19.84	203, 323	925.83 [M-H]^−^	927.48 [M+H]^+^	Saponins
8	21.29	203, 329	1099.91 [M-H]^−^	1101.37 [M+H]^+^	Saponins
9	21.69	205	967.93 [M-H]^−^	969.42 [M+H]^+^	Saponins
10	22.35	203, 322	969.86 [M-H]^−^	971.17 [M+H]^+^	Saponins
11	22.91	205	909.88 [M-H]^−^	911.42 [M+H]^+^	Saponins

^1^ Derived from the HPLC-DAD/ELSD experiments; ^2^ identified using pure reference substance; other substances are only tentatively assigned.

**Table 2:** Tentative annotation of main components and compound classes found in Herba Saniculae using HPLC-DAD/ELSD and UHPLC-ESIMS.

Peak ID ^1^	Rt. [min] ^1^	UV max [nm] ^1^	*m*/*z* [neg.]	*m*/*z* [pos.]	Putatively Assigned Compound/Compound Class
1	2.29	nd	341.31 [M-H]^−^, 387.25 [M+FA-H]^−^, 683.41 [2M-H]^−^, 729.20 [2M+FA-H]^−^, 1025.23 [3M-H]^−^, 1071.20 [3M+FA-H]^−^	365.36 [M+Na]^+^, 381.28 [M+K]^+^, 707.21 [2M+Na]^+^, 723.13 [2M+K]^+^	Sucrose ^2^ (sugars)
2	15.60	202, 242, 325	191.11 [M-H]^−^ Quinic acid, 353.29 [M-H]^−^, 707.39 [2M-H]^−^, 1061.03 [3M-H]^−^	355.24 [M+H]^+^, 377.30 [M+Na]^+^	Chlorogenic acid ^2^
3	17.24	200, 283, 324	161.08 [M-H]^−^ Glucopyranose, 359.31 [M-H]^−^ Rosmarinic acid, 521.39 [M-H_2_O-H]^−^, 539.34 [M-H]^−^	163.06 [M+H]^+^ Glucopyranose, 361.22 [M+H]^+^ Rosmarinic acid, 541.30 [M+H]^+^	Rosmarinic acid derivative
4	18.35	201, 244, 328	353.29 [M-Caffeoyl-H]^−^, 515.36 [M-H]^−^	499.36 [M-H_2_O+H]^+^, 517.15 [M+H]^+^	3,4-Dicaffeoylquinic acid ^2^
5	18.70	196, 211, 329	359.28 [M-H]^−^, 719.27 [2M-H]^−^, 1079.04 [3M-H]^−^	361.12 [M+H]^+^, 721.09 [2M+H]^+^, 1081.09 [3M+H]^+^	Rosmarinic acid ^2^
6	21.35	203, 323	1099.89 [M-H]^−^	1101.26 [M+H]^+^	Saponins
7	21.89	201	1101.91 [M-H]^−^	1085.38 [M-H_2_O+H]^+^, 1103.34 [M-+H]^+^	Saponins
8	22.37	203, 315	969.83 [M-H]^−^	953.16 [M-H_2_O+H]^+^, 971.15 [M+H]^+^	Saponins
9	22.90	196	1001.89 [M-H]^−^	1003.35 [M+H]^+^	Saponins
10	23.41	202, 322	969.85 [M-H]^−^	971.42 [M+H]^+^	Saponins

^1^ Derived from the HPLC-DAD/ELSD experiments; ^2^ identified using pure reference substance; other substances are only tentatively assigned.

**Table 3:** Tentative annotation of main components and compound classes found in Radix Cardamines en. using HPLC-DAD/ELSD and UHPLC-ESIMS.

Peak ID ^1^	Rt. [min] ^1^	UV max [nm] ^1^	*m*/*z* [neg.]	*m*/*z* [pos.]	Putatively Assigned Compound/Compound Class
1	2.29	nd	341.33 [M-H]^−^, 387.27 [M+FA-H]^−^, 683.45 [2M-H]^−^, 729.23 [2M+FA-H]^−^, 1025.26 [3M-H]^−^, 1071.23 [3M+FA-H]^−^	365.35 [M+Na]^+^, 381.32 [M+K]^+^, 707.37 [2M+Na]^+^, 723.19 [2M+K]^+^	Sucrose ^2^ (sugars)
2	4.18	196	376.26 [M-H]^−^, 753.17 [2M-H]^−^, 1129.94 [3M-H]^−^	378.24 [M+H]^+^, 755.02 [2M+H]^+^, 1132.05 [3M+H]^+^	Glucosisymbrin (glucosinolate)
3	6.41	230	404.31 [M-H]^−^, 809.20 [2M-H]^−^, 1214.00 [3M-H]^−^	406.25 [M+H]^+^, 811.07 [2M+H]^+^, 1216.04 [3M+H]^+^	Glucocleomin (glucosinolate)

^1^ Derived from the HPLC-DAD/ELSD experiments; ^2^ identified using pure reference substance; other substances are only tentatively assigned.

## Data Availability

Data are contained within the article and supplementary materials.

## Supplementary Materials

The following supporting information can be downloaded at https://www.mdpi.com/article/10.3390/plants14020266/s1: Figure S1. TLC of the extract Herba Saniculae in system B derivatized with anisaldehyde (left) and blood gelatin (right). Figure S2: UHPLC-ESIMS base peak chromatogram in positive mode (upper) and in negative mode (middle) and CAD chromatogram (lower) of Radix Saniculae. Figure S3: UHPLC-ESIMS base peak chromatogram in positive mode (upper) and in negative mode (middle) and CAD chromatogram (lower) of Herba Saniculae. Figure S4: UHPLC-ESIMS base peak chromatogram in positive mode (upper) and in negative mode (middle) and CAD chromatogram (lower) of Radix Cardamines enneaphyllos. Table S1: UV spectra and mass spectra (negative mode upper spectrum; positive mode lower spectrum) of main peaks found in the extracts of Radix Saniculae. Table S2: UV spectra and mass spectra (negative mode upper spectrum; positive mode lower spectrum) of main peaks found in the extracts of Herba Saniculae. Table S3: UV spectra and mass spectra (negative mode upper spectrum; positive mode lower spectrum) of main peaks found in the extracts of Radix Cardamines enneaphyllos. Table S4. GC-MS data detected for sisymbrin and cleomin in fresh *Cardamine enneaphyllos* roots.
