# Supplementary material for: “Radix Saniculae”: Phytochemical Characterization and Potential Adulteration of an Austrian Traditional Wound-Healing Agent
Source: Plants (Basel). 2025 Jan 18;14(2):266. doi: 10.3390/plants14020266 (PMC11768901; doi:10.3390/plants14020266)
Supplement: Supplementary file 1 [file plants-14-00266-s001.zip › plants-3354799-supplementary.pdf]

Supplementary Materials:

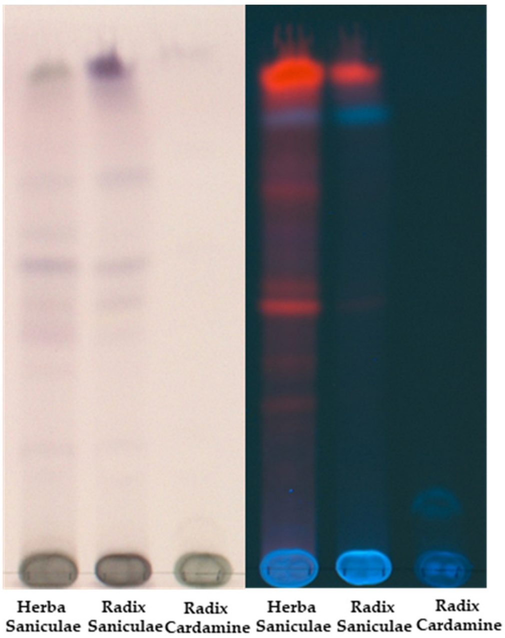

Figure S1. TLC comparison of the extracts in system A after derivatization with anisaldehyde (left) at visible light, and without derivatization at 365 nm (right).

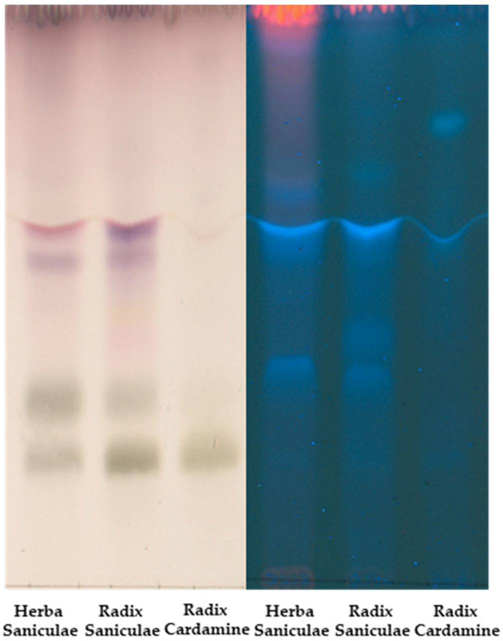

Figure S2. TLC comparison of the extracts in system B after derivatization with anisaldehyde (left) at visible light, and without derivatization at 365 nm (right).

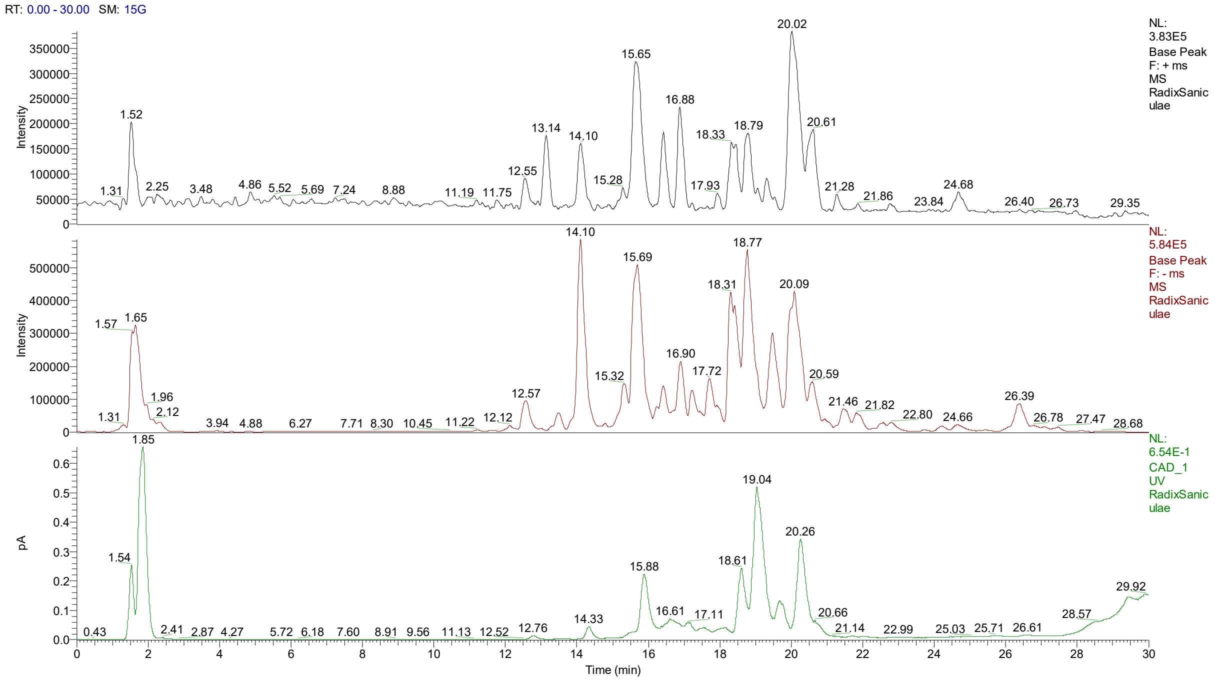

Figure S3. UHPLC-ESIMS base peak chromatogram in positive mode (upper), in negative mode (middle) and CAD chromatogram (lower) of Radix Saniculae.

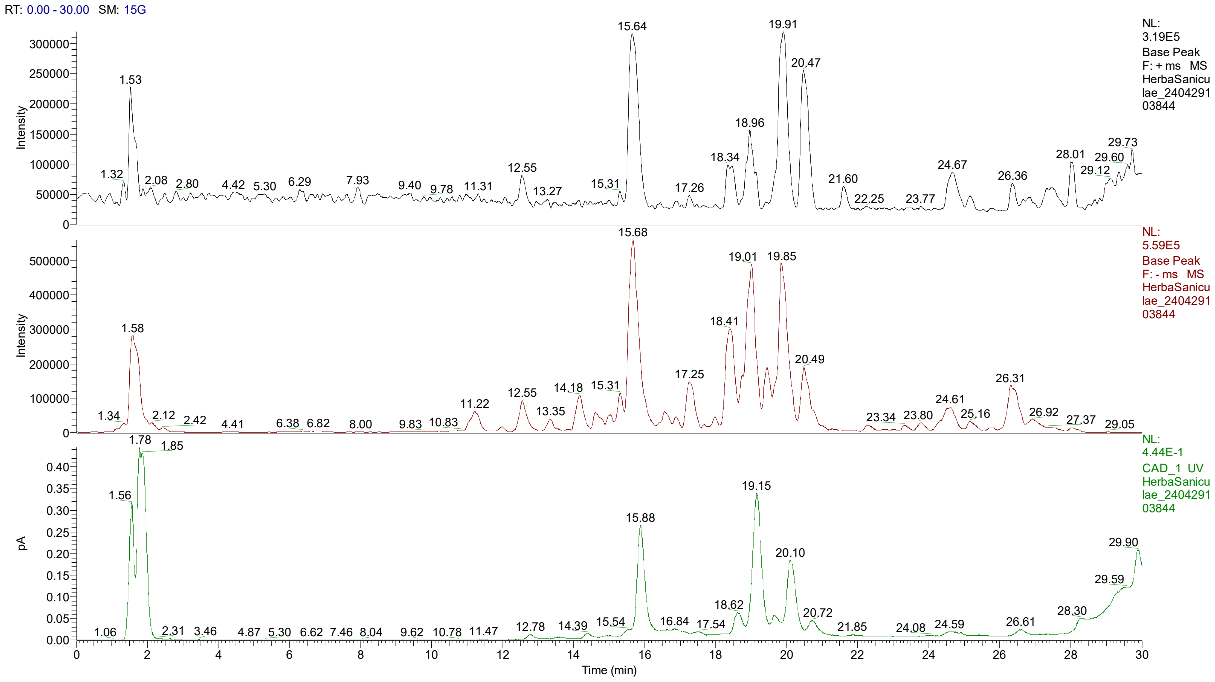

Figure S4. UHPLC-ESIMS base peak chromatogram in positive mode (upper), in negative mode (middle) and CAD chromatogram (lower) of Herba Saniculae.

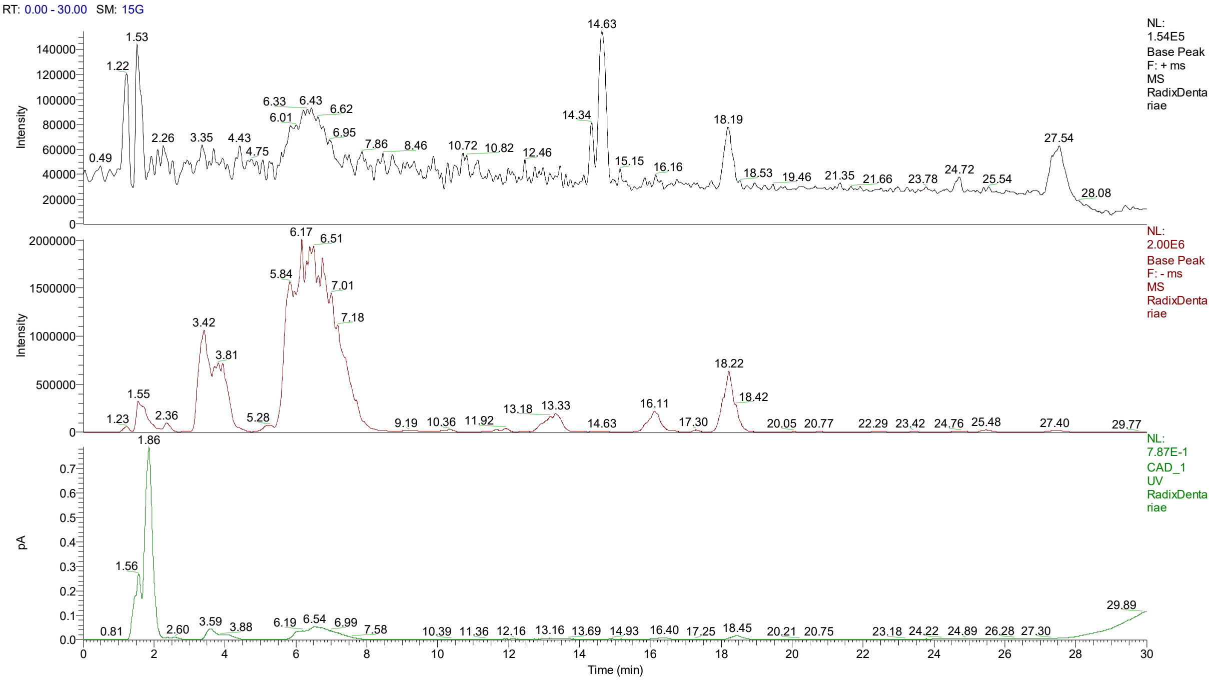

Figure S5. UHPLC-ESIMS base peak chromatogram in positive mode (upper), in negative mode (middle) and CAD chromatogram (lower) of Radix Cardamines enneaphyllos.

Table S1. UV spectra and mass spectra (negative mode upper spectrum, positive mode lower spectrum) of main peaks found in the extracts of Radix Saniculae.

Peak Nr.	UV spectrum	ESIMS
1	nd	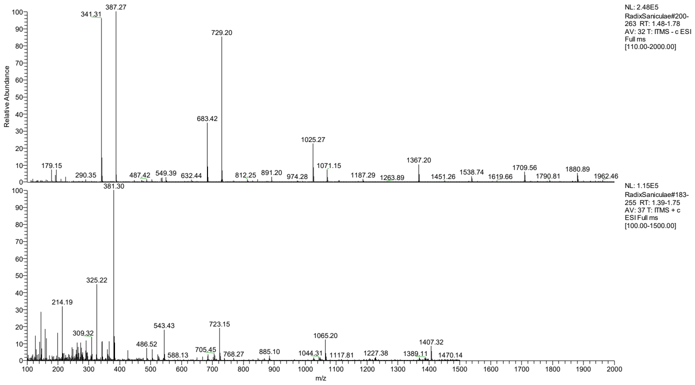
2	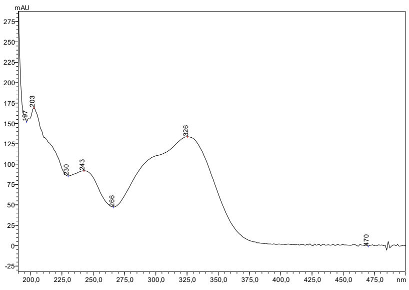	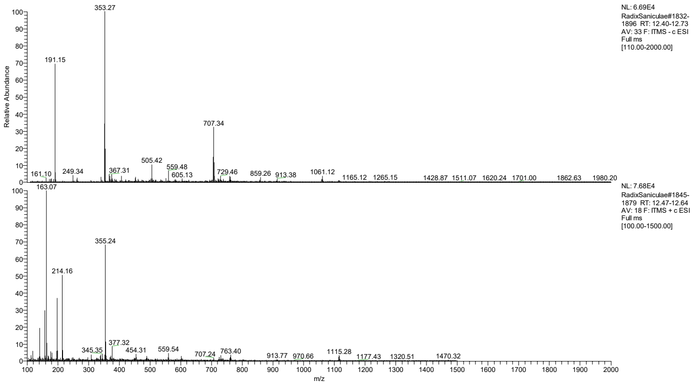

3

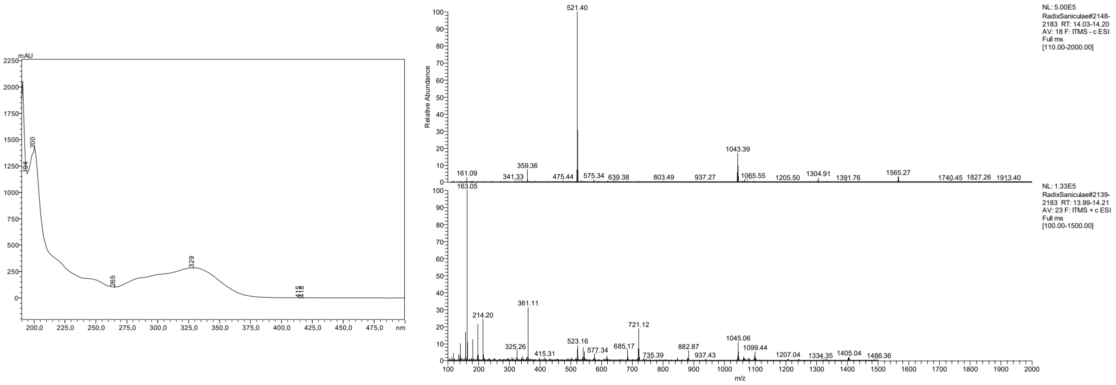

mAU
nm
Relative Abundance
m/z
NL: 5.00E5
RadixSaniculae#2148-2183 RT: 14.03-14.20
AV: 18 F: ITMS - c ESI
Full ms
[110.00-2000.00]
521.40
1043.39
NL: 1.33E5
RadixSaniculae#2139-2183 RT: 13.99-14.21
AV: 23 F: ITMS + c ESI
Full ms
[100.00-1500.00]
163.05
361.11
214.20
721.12

4

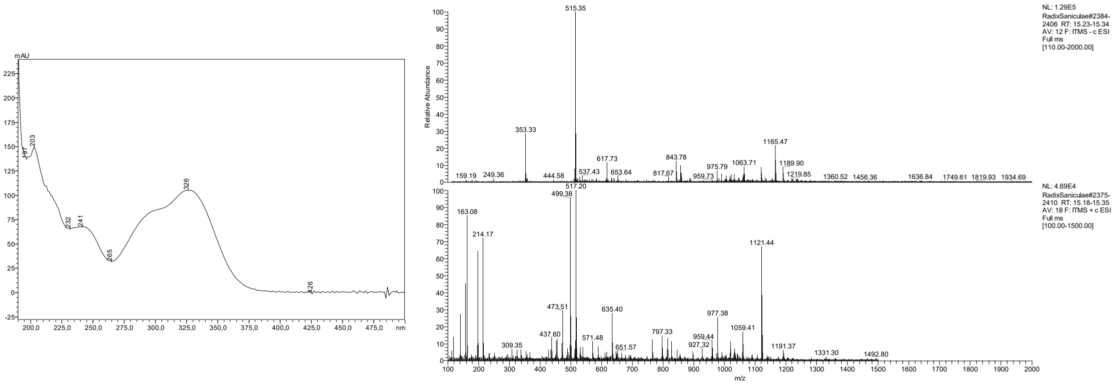

mAU
nm
Relative Abundance
m/z
NL: 1.29E5
RadixSaniculae#2384-2406 RT: 15.23-15.34
AV: 12 F: ITMS - c ESI
Full ms
[110.00-2000.00]
515.35
353.33
1165.47
NL: 4.69E4
RadixSaniculae#2375-2410 RT: 15.18-15.35
AV: 18 F: ITMS + c ESI
Full ms
[100.00-1500.00]
517.20
499.38
163.08
214.17
1121.44

5

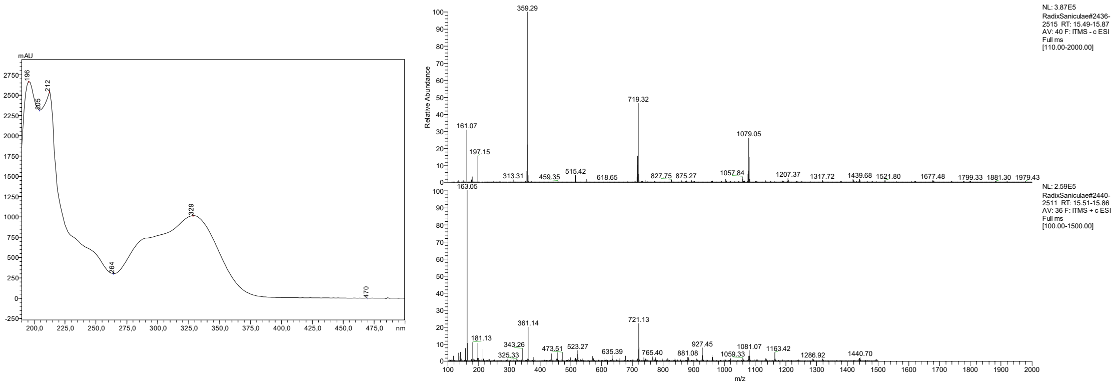

mAU
nm
Relative Abundance
m/z
NL: 3.87E5
RadixSaniculae#2436-2515 RT: 15.49-15.87
AV: 40 F: ITMS - c ESI
Full ms
[110.00-2000.00]
359.29
719.32
1079.05
NL: 2.59E5
RadixSaniculae#2440-2511 RT: 15.51-15.86
AV: 36 F: ITMS + c ESI
Full ms
[100.00-1500.00]
163.05
361.14
721.13

6

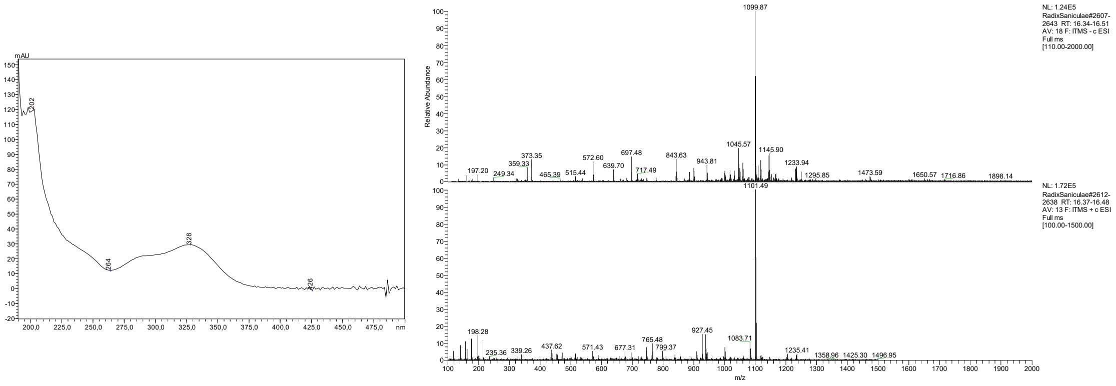

mAU
nm
Relative Abundance
m/z
NL: 1.24E5
RadixSaniculae#2607-2643 RT: 16.34-16.51
AV: 18 F: ITMS - c ESI
Full ms
[110.00-2000.00]
1099.87
1045.57
1145.90
NL: 1.72E5
RadixSaniculae#2612-2638 RT: 16.37-16.48
AV: 13 F: ITMS + c ESI
Full ms
[100.00-1500.00]
1101.49
927.45

7

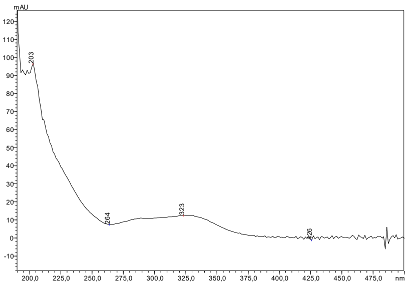

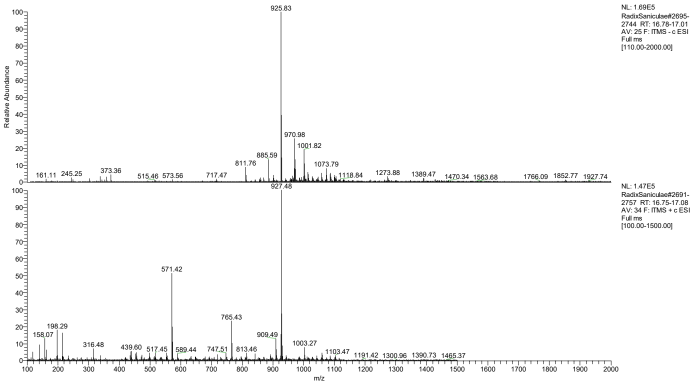

8

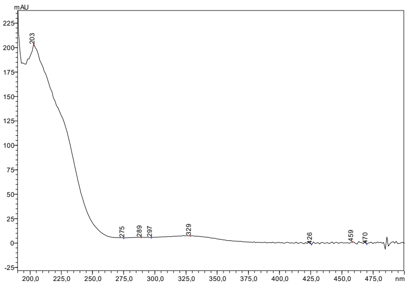

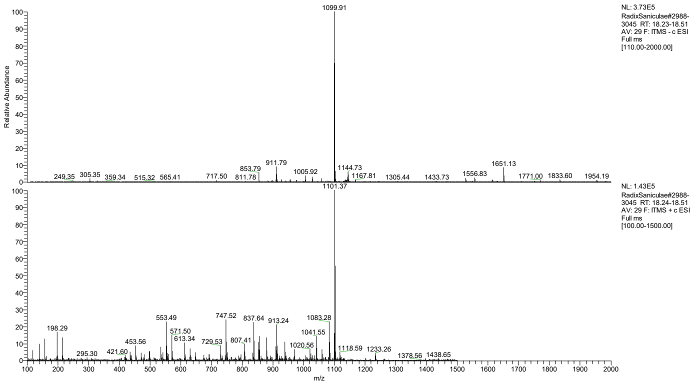

9

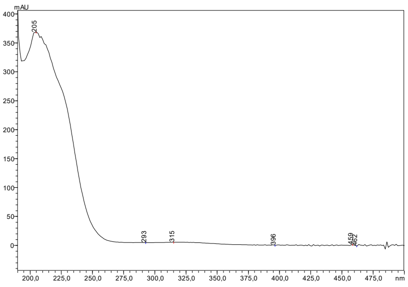

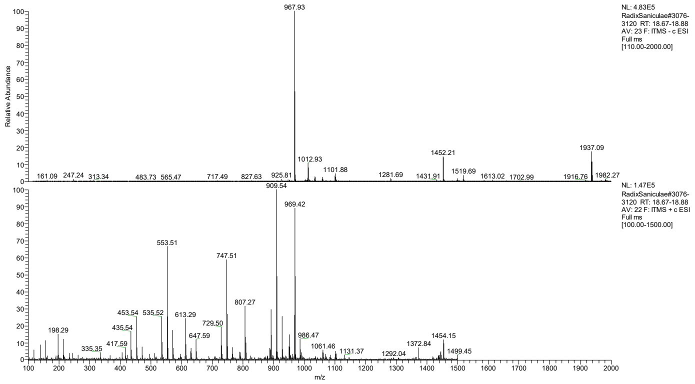

10

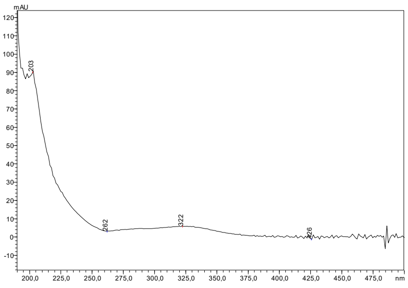

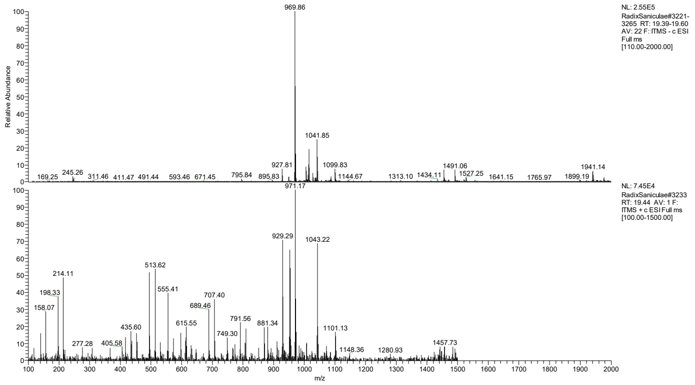

11	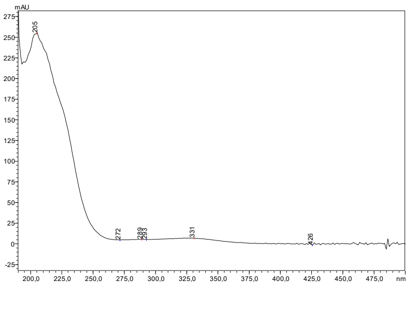	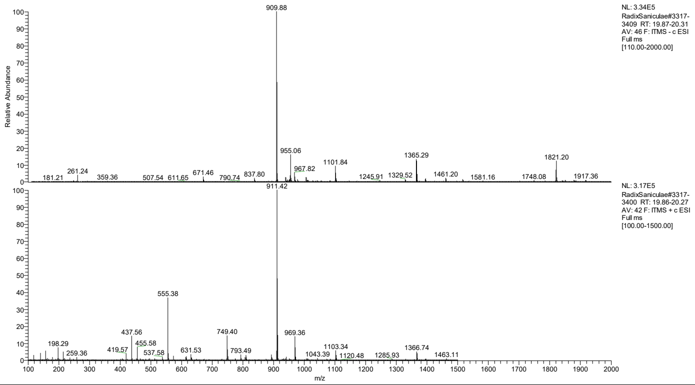

Table S2. UV spectra and mass spectra (negative mode upper spectrum, positive mode lower spectrum) of main peaks found in the extracts of Herba Saniculae.

Peak Nr.	UV spectrum	ESIMS
1	nd	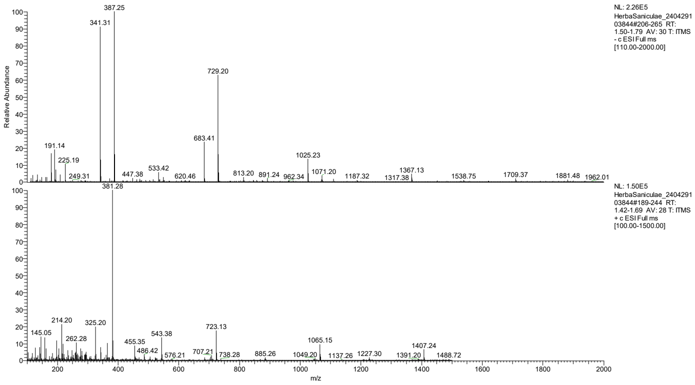
2	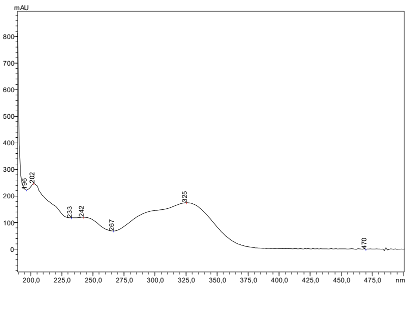	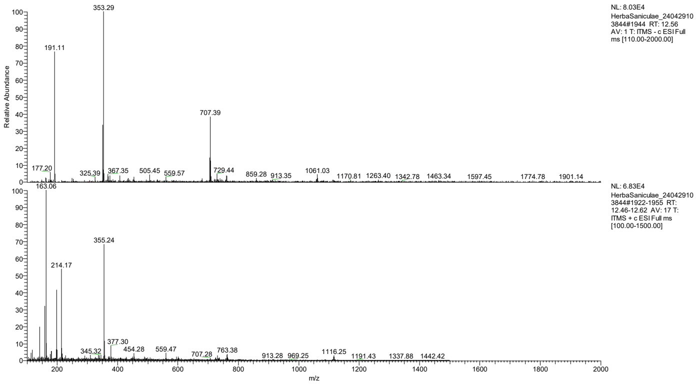
3	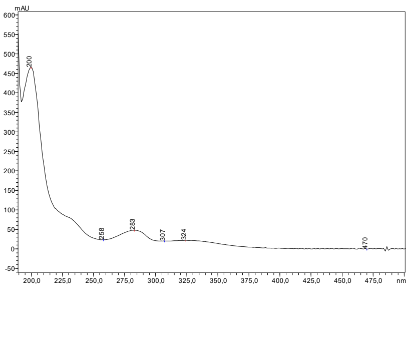	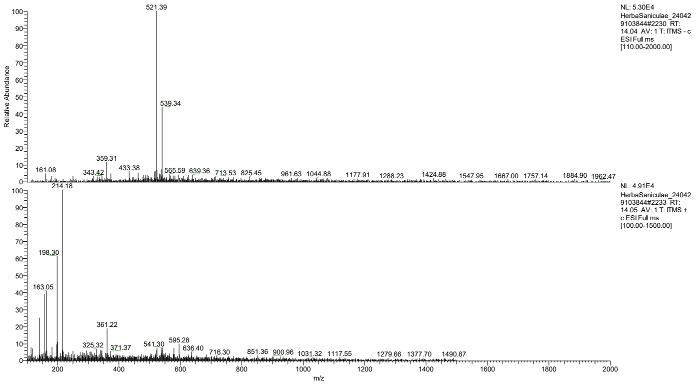

4

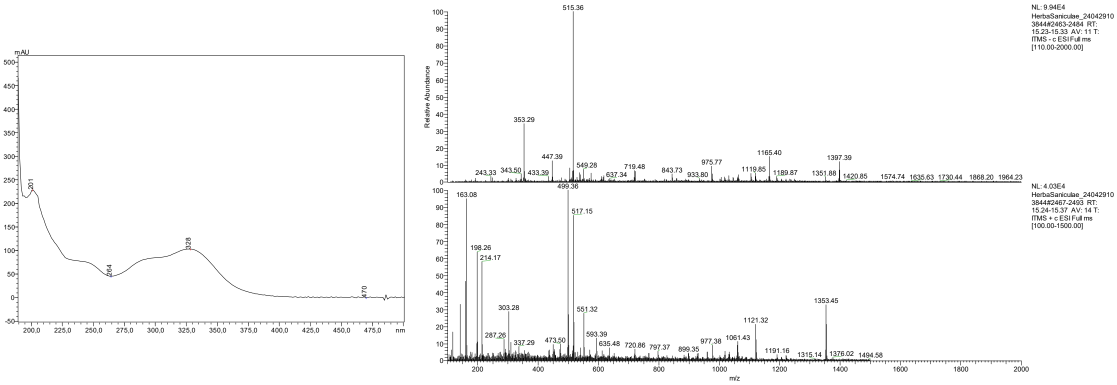
mAU
nm
Relative Abundance
m/z
NL: 9.94E4
HerbaSaniculae_24042910
3844#2463-2484 RT:
15.23-15.33 AV: 11 T:
ITMS - c ESI Full ms
[110.00-2000.00]
NL: 4.03E4
HerbaSaniculae_24042910
3844#2467-2493 RT:
15.24-15.37 AV: 14 T:
ITMS + c ESI Full ms
[100.00-1500.00]

5

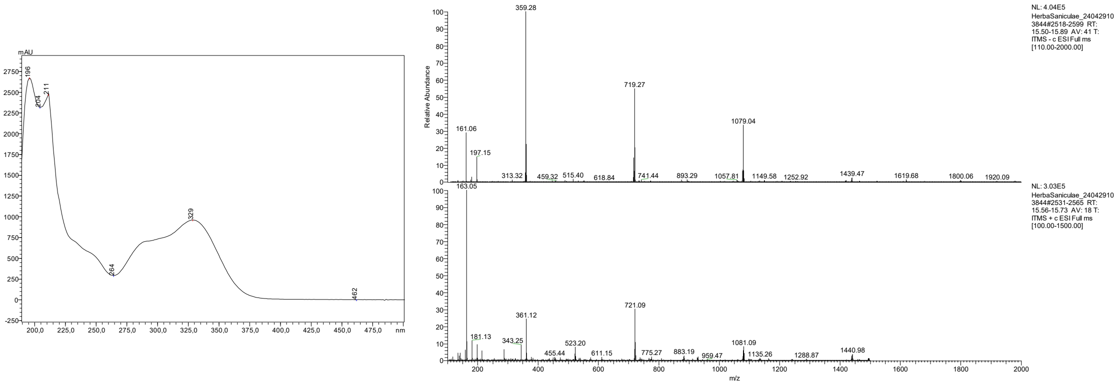
mAU
nm
Relative Abundance
m/z
NL: 4.04E5
HerbaSaniculae_24042910
3844#2518-2599 RT:
15.50-15.89 AV: 41 T:
ITMS - c ESI Full ms
[110.00-2000.00]
NL: 3.03E5
HerbaSaniculae_24042910
3844#2531-2565 RT:
15.56-15.73 AV: 18 T:
ITMS + c ESI Full ms
[100.00-1500.00]

6

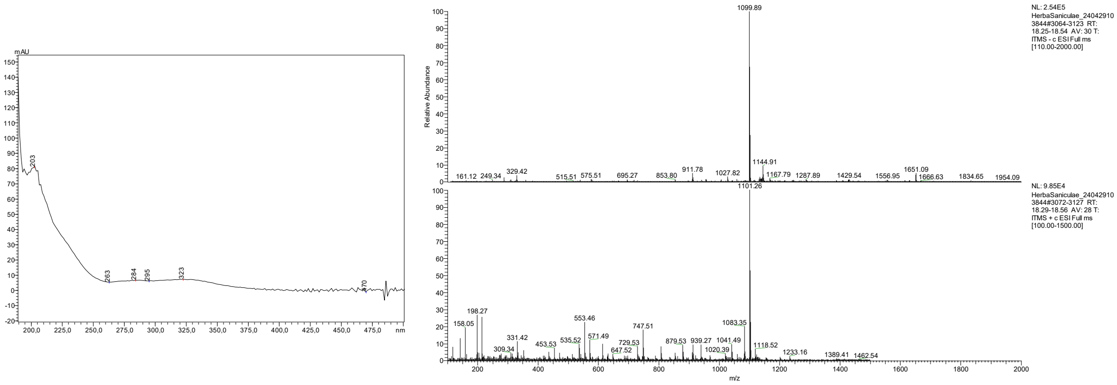
mAU
nm
Relative Abundance
m/z
NL: 2.54E5
HerbaSaniculae_24042910
3844#3064-3123 RT:
18.25-18.54 AV: 30 T:
ITMS - c ESI Full ms
[110.00-2000.00]
NL: 9.85E4
HerbaSaniculae_24042910
3844#3072-3127 RT:
18.29-18.56 AV: 28 T:
ITMS + c ESI Full ms
[100.00-1500.00]

7

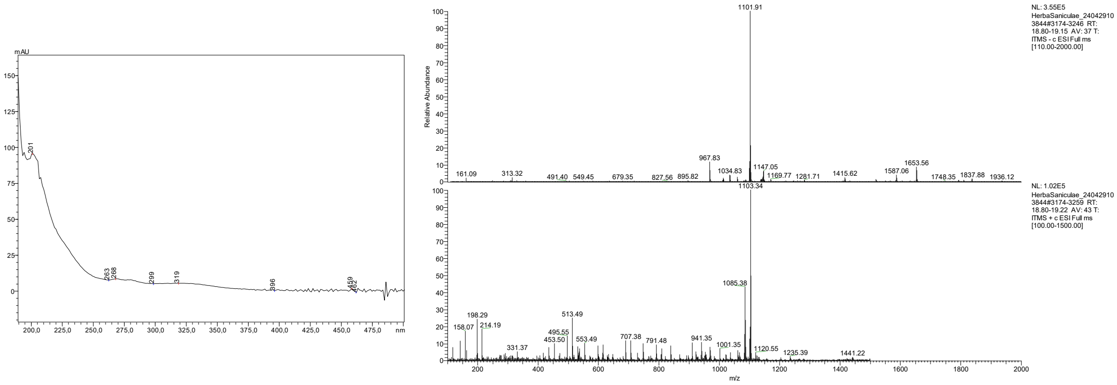
mAU
nm
Relative Abundance
m/z
NL: 3.55E5
HerbaSaniculae_24042910
3844#3174-3246 RT:
18.80-19.15 AV: 37 T:
ITMS - c ESI Full ms
[110.00-2000.00]
NL: 1.02E5
HerbaSaniculae_24042910
3844#3174-3259 RT:
18.80-19.22 AV: 43 T:
ITMS + c ESI Full ms
[100.00-1500.00]

8	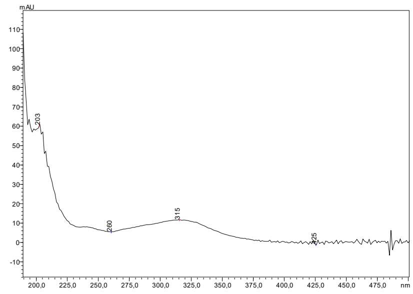	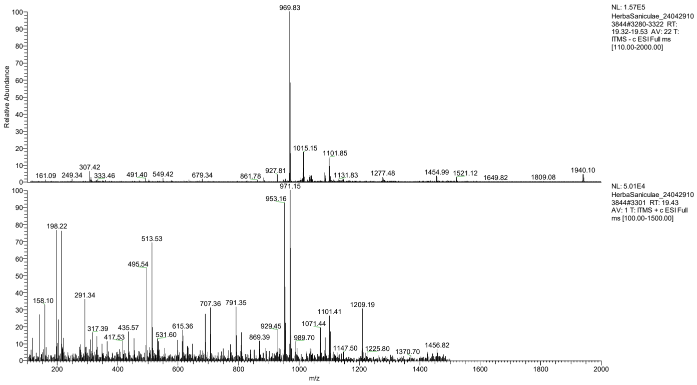
9	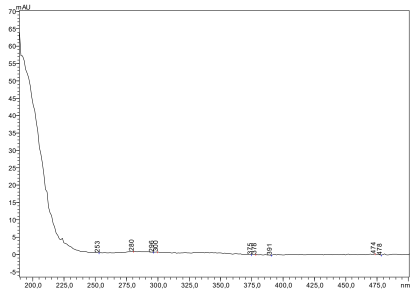	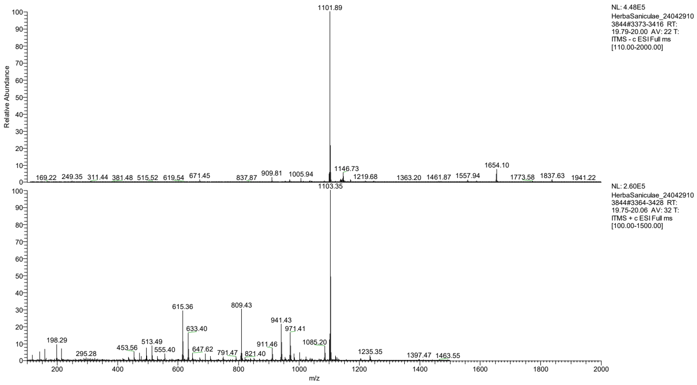
10	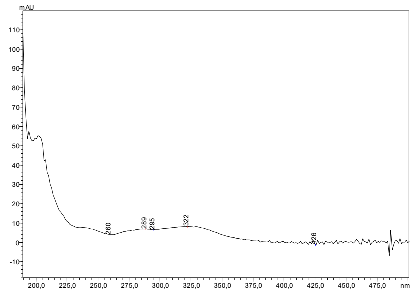	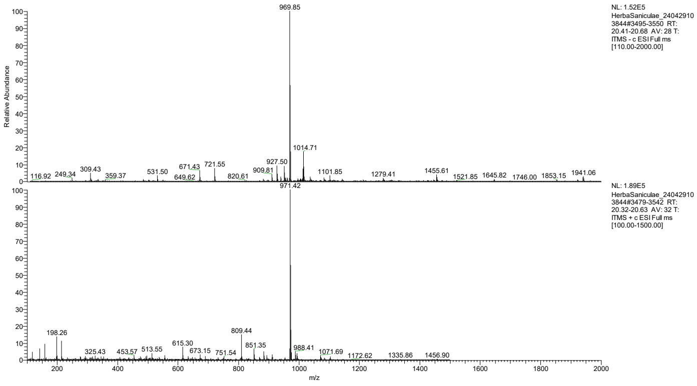

Table S3. UV spectra and mass spectra (negative mode upper spectrum, positive mode lower spectrum) of main peaks found in the extracts of Radix Cardamines enneaphyllos.

Peak Nr.	UV spectrum	ESIMS
1	nd	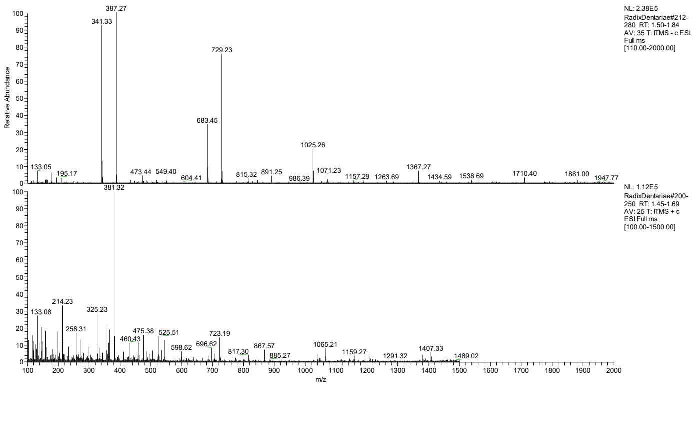

2

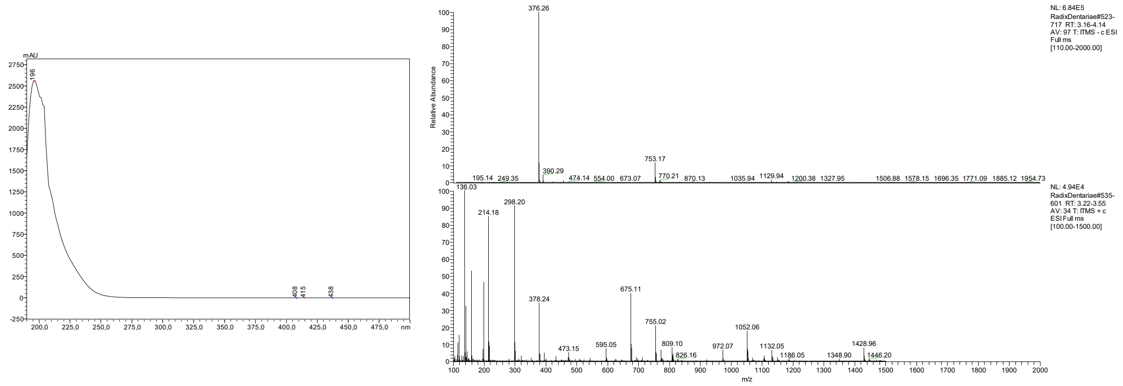

3

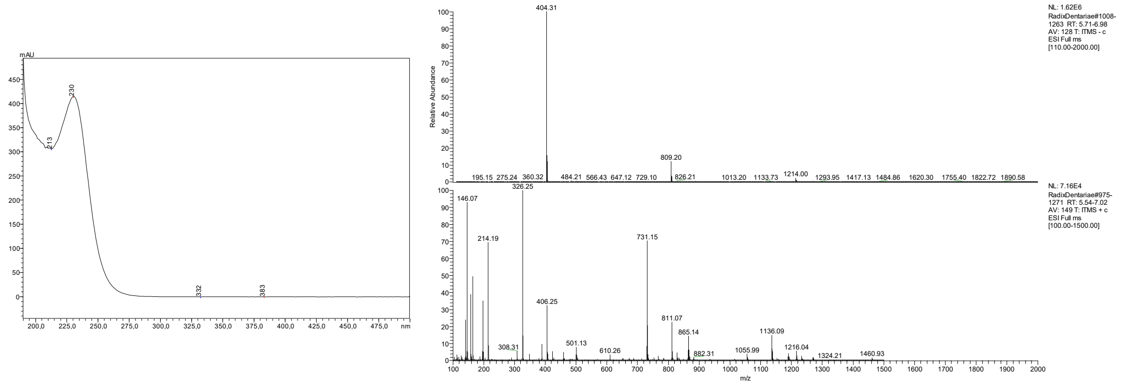

Table S4. GC-MS data detected for sisymbrin and cleomin in fresh *Cardamine enneaphyllos* roots.

Peak Nr.	Rt. [min][1]	Detected masses	Molecular weight [g/mol]	Compound name
1	25.72	117 (Molecular Ion Peak)<br>102 (- Methyl)	117	Sisymbrin
2	28.46	145 (Molecular Ion Peak)<br>130 (- Methyl)<br>116 (-Ethyl)	145	Cleomin
